# Impaired fasting glucose and major adverse cardiovascular events by hypertension and dyslipidemia status: the Golestan cohort study

**DOI:** 10.1186/s12872-020-01390-8

**Published:** 2020-03-05

**Authors:** Nahid Hashemi Madani, Faramarz Ismail-Beigi, Hossein Poustchi, Mahdi Nalini, Sadaf G. Sepanlou, Mojtaba Malek, Mohammad Amin Abbasi, Alireza Khajavi, Mohammad E. Khamseh, Reza Malekzadeh

**Affiliations:** 1 Endocrine Research Center, Institute of Endocrinology and Metabolism, Iran University of Medical Sciences (IUMS), No. 10, Firoozeh St, Valiasr Ave, Vali-asr Sq, Tehran, Iran; 2 Case Western Reserve University, School of Medicine, Cleveland, USA; 3 Liver and Pancreatobiliary Diseases Research Center, Digestive Diseases Research Institute, Tehran University of Medical Sciences, Tehran, Iran; 4 Digestive Disease Research Center, Digestive Disease Research Institute, Tehran University of Medical Sciences, Tehran, Iran; 5 Research Center for Prevention of Cardiovascular Diseases, Institute of Endocrinology and Metabolism, Iran University of Medical Sciences (IUMS), Tehran, Iran; 6 Student Research Committee, Faculty of Paramedical Sciences, Shahid Beheshti University of Medical Science, Tehran, Iran

**Keywords:** Dyslipidemia (DLP), Hypertension (HTN), Impaired fasting glucose (IFG), Major adverse cardiovascular events (MACE), Pre-diabetes (pre-DM)

## Abstract

**Background:**

Whether pre-diabetes in the absence of hypertension (HTN) or dyslipidemia (DLP) is a risk factor for occurrence of major adverse cardiovascular events (MACE) is not fully established. We investigated the effect of impaired fasting glucose (IFG) alone and in combination with HTN, DLP or both on subsequent occurrence of MACE as well as individual MACE components.

**Methods:**

This longitudinal population-based study included 11,374 inhabitants of Northeastern Iran. The participants were free of any cardiovascular disease at baseline and were followed yearly from 2010 to 2017. Cox proportional hazard models were fitted to measure the hazard of IFG alone or in combination with HTN and DLP on occurrence of MACE as the primary endpoint.

**Results:**

Four hundred thirty-seven MACE were recorded during 6.2 ± 0.1 years follow up. IFG alone compared to normal fasting glucose (NFG) was not associated with an increase in occurrence of MACE (HR, 0.87; 95% CI, 0.19–4.02; p, 0.854). However, combination of IFG and HTN (HR, 2.88; 95% CI, 2.04–4.07; p, 0.000) or HTN + DLP (HR, 2.98; 95% CI, 1.89–4.71; p, 0.000) significantly increased the risk for MACE. Moreover, IFG + DM with or without HTN, DLP, or both was also associated with an increase in the incidence of MACE.

**Conclusion:**

IFG, per se, does not appear to increase hazard of MACE. However, IFG with HTN or HTN + DLP conferred a significant hazard for MACE in an incremental manner. Moreover, IFG without HTN, adjusted for DLP, can be associated with an increase in the risk for CVD- death.

## Background

Pre-diabetes (pre-DM) is often taken as a warning sign. Individuals with pre-DM have a glycemic state that is higher than normal but not high enough to be classified as type 2 diabetes (T2DM). The pre-diabetes state is characterized by impaired fasting glucose (IFG), impaired glucose tolerance (IGT), or HbA1c of 39 mmol/mol (5.7%) to 46 mmol/mol (6.4%) [1]. The significance of pre-DM is the associated risk for progression to T2DM which is disproportionately greater at the higher end of the pre-DM range and with the combined presence of IFG and IGT [1]. Pre-DM and T2DM are parts of a continuous spectrum that share in their pathophysiology, and are associated with a common phenotype that includes obesity, hypertension (HTN), and dyslipidemia (DLP) [2]. It is well established that aggregation of the traditional cardiovascular (CV) risk factors such as HTN and DLP in patients with T2DM is associated with the increased risk of cardiovascular disease (CVD) and events [3, 4]. Furthermore, insulin resistance per se that is present in the vast majority of individuals with T2DM is associated with the increased risk of CVD independent of other CV risk factors [5, 6].

In addition to T2DM, pre-DM is also associated with an increased risk of CVD [7] [8]. Considering that traditional CV risk factors are frequently present in individuals with pre-diabetes, the question arises that to what extent is the effect of pre-DM on CVD risk mediated by having pre-DM alone or by the associated risk factors [9, 10]. Since some studies did not fully adjust for concomitant CV risk factors, their findings should be interpreted with caution. Hence, whether pre-DM or IFG alone in the absence of HTN or DLP or their combination carries increased risk for CVD has remained an unanswered issue.

Therefore, we conducted a study using data from Golestan Cohort Study (GCS) to investigate whether pre-diabetes in the absence of HTN or DLP is a risk factor for occurrence of major adverse cardiovascular events (MACE) as well as its individual components. We also examined the risk for MACE conferred by diabetes in the presence or absence of HTN or DLP. We further did the analysis in which patients with IFG and DM are considered together in comparison with patients with NFG.

## Methods

### Study design and population

We extracted data from GCS a community-based prospective cohort study that was launched in Northeastern Iran [11]. The primary aim of GCS was to investigate risk factors of esophageal cancer in this region. The GCS enrolled temporary residents from the rural or urban areas of this region who aged 40–75 years, and were free from any upper gastrointestinal cancer. Participants in this cohort were also followed for CVD outcomes. In this analysis, we included data from a total number of 11,374 participants who had no current or previous history of any known CVD events and had complete information on fasting blood sugar (FBS), blood pressure (BP), and lipid profile at the time of recruitment (2010–2012). The ethical review committees of the Digestive Disease Research Institute of Tehran University of Medical Sciences, the US National Cancer Institute, and the International Agency for Research on Cancer approved the study protocol. Before interview, the written informed consent was obtained from each participant.

Baseline data were collected during an interview by a trained general physician, either in the local language (Turkmen) or in the national formal language (Persian), depending on the participant’s preference. Questions were asked about the age, residence area (urban or rural), smoking status, alcohol consumption, history of any heart disease, or any kind of cancer. Height, weight, waist and hip circumference were measured using standard methods, and body mass index (BMI) was calculated. BP was measured twice, with a 10-min interval, from both arms in the sitting position using Richter auscultator sphygmomanometers. A trained technician collected Blood samples (10 ml). In the urban area, the samples were immediately processed in the central laboratory at the Golestan Cohort Study Center. In the rural areas, blood samples were kept in refrigerators (+4C), until they were transferred in cooling boxes to the central laboratory; the maximum duration between blood collection and final processing was 8 h. FBS, triglyceride (TG), cholesterol, and high density lipoprotein (HDL) were measured enzymatically in all individuals [11].

### Follow up

Participants were followed up by annual phone calls from their date of recruitment until the first occurrence of any CVD event, death from any cause, or end of the study (March 2017). Each cohort member was also instructed at the time of enrollment to contact the team in case of certain conditions such as hospitalization or development of a new major disease. These contacts were registered and subsequently followed by a staff member. In case a death, cancer or any CVD event was reported, the GCS team visited the participant’s home and the medical centers in which any major diagnostic or therapeutic procedures were done. The team collected all clinical reports, pathology reports and hospital records. Follow-up assessed at the end of the Golestan study was successful for over 99% of the study participants. If a death was identified, a general practitioner visited the home of the deceased. Causes of death were ascertained through verbal interview and investigation of medical documents [12]. Using the collected documents, two internists independently determined the cause of death with ICD-10 codes. If the two were concordant, a diagnosis was made. Otherwise, a third senior internist determined the cause of death.

### Definition of IFG, DM, HTN, and DLP

IFG was diagnosed when a participant did not have DM, but had an FBS of 5.6 to < 7.0 mmol/L, according to the American Diabetes Association criteria (ADA) [1]. DM was defined as a self-reported diagnosis, or being treated with glucose lowering drugs, or those with FBS ≥ 7 mmol/L. Normal fasting glucose (NFG) was defined as FBS < 5.6 mmol/L. Hypertension was defined as self-reported hypertension and currently taking antihypertensive drugs, or systolic blood pressure (SBP) ≥140 mmHg or diastolic blood pressure (DBP) ≥ 90 mmHg based on the JNC-7 criteria [13]. DLP was defined as fasting TG ≥1.69 mmol/L, or total cholesterol ≥6.20 mmol/L, or HDL < 1.29 mmol/L in women and < 1.03 mmol/L in men, or receiving lipid lowering medications.

### Definition of MACE

The primary outcome in the current analysis was the first occurrence of any component of the 3-point MACE defined as non-fatal myocardial infarction (MI), non-fatal stroke, and CVD death. CVD death composed of death due to coronary heart disease, stroke, or heart failure.

### Statistical analysis

Data are presented as means (SE) and numbers (percentage). In all cases, final analysis was based on presence or absence of a risk factor (or factors) in the DM, IFG, and NGT groups using fully adjusted models. Student t-test and Pearson χ2 (or Fisher Exact) tests were used to compare continuous and categorical variables, respectively. Risk analysis was conducted in patients with IFG, and Poisson regression model was used to identify predictors of MACE (non-fatal MI, and non-fatal stroke, and CVD death). Regarding the time to event for MACE and its components, Kaplan-Meyer method was used to estimate the survival probabilities, and Cox proportional hazard models were fitted to measure the hazard ratios. Statistical analyses were performed with Stata software for Windows version 13.0. Values of *p* < 0.05 was considered statistically significant.

## Results

### Baseline characteristics

In this study, we included 11,374 participants from the Northern provinces of Iran who were free of any CVD events at the time of recruitment. Among the cohort 1543 (13.6%) individuals had DM, 2410 (21.2%) had IFG, and 7421 (65.3%) had normal fasting glucose (NFG).

Baseline characteristics of the study population are demonstrated in Table 1. The mean age of the participants was 56.2 (0.1) years and 47.5% of them were men. There was a significant difference in the age, sex, smoking status, percentage of statin users, BMI, waist circumference (WC), waist-to-hip ratio (W/H), SBP, DBP, FBS, TG, Chol, low density lipoprotein (LDL), percentage of subjects with DLP or HTN among the studied groups (*p* < 007, all). Moreover, the comparison between NFG and IFG showed a significantly higher prevalence of HTN (44.2 vs. 34.7%; p, 0.000) and DLP (49.9 vs. 39.7%; p, 0.000) in IFG group. Moreover, compared to NFG group, the IFG group were older and had a significantly higher level of BMI, WC, SBP, DBP, TG, cholesterol, and LDL (*p* = 0.000, all). Participants with IFG were more likely to use a statin medication than did the NFG group (3.7 vs. 2.7%, p, 0.007).

**Table 1:** Baseline characteristics of the participants according to the glycemic status

Variables	Total (*N* = 11,374)	NFG (*N* = 7421)	IFG (*N* = 2410)	DM(*N* = 1543)	*p*-value
Age (yr.)*	56.2 (0.1)	55.8 (0.1)	56.7 (0.2)	57.4 (0.2)	0.000
Sex (%M)	47.5%	48.4%	47.3%	43.2%	0.001
Smoking (%)					0.000
Past	7.9%	7.8%	8.8%	7.2%	
Current	8.3%	9.2%	8.1%	4.3%	
Never	83.8%	83.0%	83.1%	88.5%	
Alcohol (%)	2.7%	2.7%	2.6%	3.1%	0.7
Statin (%)†	4.2%	2.7%	3.7%	12.5%	0.007
BMI (kg/m^2^) *	27.1 (0.1)	26.5 (0.1)	27.6 (0.1)	29.2 (0.1)	0.000
WC (cm) *	94.6 (0.1)	92.5 (0.2)	96.2 (0.3)	101.9 (0.3)	0.000
W/H*	0.43 (0.001)	0.43 (0.001)	0.44 (0.002)	0.47 (0.002)	0.000
SBP (mmHg) *	125.7 (0.2)	123.9 (0.2)	128.0 (0.4)	130.7 (0.5)	0.000
DBP (mmHg) *	77.6 (0.1)	76.8 (0.1)	78.4 (0.3)	80.0 (0.3)	0.000
FBS (mmol/L) *	104.8 (0.4)	87.7 (0.1)	108.1 (0.1)	181.3 (1.8)	0.000
TG (mmol/L) *	138.7 (0.9)	126.9 (1.0)	145.5 (1.9)	185.2 (3.8)	0.000
Chol (mmol/L) *	203.5 (0.5)	200.3 (0.7)	207.1 (0.9)	213.3 (1.2)	0.000
LDL (mmol/L) *	118.2 (0.5)	116.4 (0.7)	121.1 (1.0)	122.3 (1.3)	0.000
HDL (mmol/L)	61.5 (0.6)	61.2 (0.3)	61.4 (0.8)	62.6 (3.7)	0.7
DLP %*	5189 (45.4%)	2945 (39.7%)	1202 (49.9%)	1040 (67.4%)	0.000
HTN %*	4517 (39.6%)	2578 (34.7%)	1064 (44.2%)	860 (55.7%)	0.000

Data are presented as mean (standard error) and number (percentage)

Comparing NFG and IFG groups: * *p* = 0.000, †*p* = 0.007

*BMI* Body mass index, *WC* Waist circumference, *W/H* Waist to hip ratio, *SBP* Systolic blood pressure, *DBP* Diastolic blood pressure, *FBS* Fasting blood glucose, *TG* Triglyceride, *Chol* Cholesterol, *LDL* Low density lipoprotein, *HDL* High density lipoprotein, *DLP* Dyslipidemia, *HTN* Hypertension

### IFG and CVD outcomes

Over a mean follow up of 6.2 (± 0.1) years, 437 MACE (283 CVD deaths, 57 non-fatal MI, and 97 non-fatal strokes) were reported. CVD death comprised of 155 fatal MI, 101 fatal strokes, and 27 deaths due to heart failure.

Table 2 shows HRs and 95% CI of MACE according to glycemic status, HTN, and DLP. The occurrence of MACE was not significantly different between those with IFG but without HTN or DLP compared to NFG participants who also did not have HTN or DLP (fully adjusted HR, 1.07; 95% CI, 0.60–1.90; p, 0.817). However, compared to normoglycemic subjects without HTN or DLP, the hazard for incidence of MACE increased in normoglycemic participants with HTN + DLP (fully adjusted HR, 2.67; 95% CI, 1.79–3.98; p, 0.000), IFG + HTN + DLP (fully adjusted HR, 2.98; 95% CI, 1.89–4.71; p, 0.000), and diabetic patients in the presence or absence of HTN and DLP (fully adjusted HR, 4.63; 95% CI, 3.03–7.08; p, 0.000) and (fully adjusted HR, 4.51; 95% CI, 2.65–7.68; p, 0.000), respectively. Moreover, to compare dysglycemic subjects with normoglycemic counterparts, we pooled data from IFG + DM. The results showed IFG + DM in the presence or absence of HTN and DLP was also significantly associated with an increase in the risk of MACE, (fully adjusted HR, 3.64; 95% CI, 2.50–5.31; p, 0.000) and (fully adjusted HR, 1.85; 95% CI, 1.20–2.85; p, 0.005), respectively. When participants were categorized based on glycemic status with the presence or absence of HTN alone, individuals with IFG without HTN did not increase the hazard of MACE compared to NFG without HTN (fully adjusted HR, 1.20; 95% CI, 0.78–1.85; p, 0.400). However, there was a significant increase in the hazard of MACE in the normoglycemic individuals with HTN (fully adjusted HR, 2.68; 95% CI, 2.01–3.56; p, 0.000), IFG + HTN (fully adjusted HR, 2.88; 95% CI, 2.04–4.07; p, 0.000), and diabetic participants without and with HTN (fully adjusted HR, 4.26; 95% CI, 2.95–6.16; p, 0.000) and (fully adjusted HR, 4.38; 95% CI, 3.10–6.19; p, 0.000), respectively. Furthermore, IFG + DM in the absence or presence of HTN was significantly associated with the risk of MACE, (fully adjusted HR, 2.15; 95% CI, 1.57–2.95; p, 0.000) and (fully adjusted HR, 3.38; 95% CI, 2.51–4.54; p, 0.000), respectively. HRs for MACE was also evaluated according to glycemic status in the presence or absence of DLP. Compared to the normoglycemic subjects without DLP (NFG/−DLP), IFG/+ DLP and IFG/− DLP did not increase the hazard of MACE (fully adjusted HR, 1.30; 95% CI, 0.92–1.84; p, 0.138) and (fully adjusted HR, 1.08; 95% CI, 0.76–1.53; p, 0.680), respectively. On the contrary, DM/+DLP and DM/− DLP were significantly associated with an increase in the hazard of MACE (fully adjusted HR, 2.48; 95% CI, 1.83–3.35; p, 0.000) and (fully adjusted HR, 2.35; 95% CI, 1.62–3.41; p, 0.000), respectively. Moreover, dysglycemic individuals (IFG+ DM) in the absence or presence of DLP significantly increased the hazard of MACE (fully adjusted HR, 1.44; 95% CI, 1.08–1.91; p, 0.012) and (fully adjusted HR, 1.80; 95% CI, 1.39–2.34; p, 0.000), respectively.

**Table 2:** Cox regression models predicting MACE according to glycemic status, HTN, and DLP

	HR (95% CI) for incident MACE		
Category	Unadjusted model	Adjusted model 1	Adjusted model 2^a^
NFG/−HTN/−DLP	1.0	1.0	1.0
NFG/+HTN/+DLP	2.81 (1.92–4.11) *P* = 0.000	2.59 (1.76–3.80) *P* = 0.000	2.67 (1.79–3.98) *P* = 0.000
IFG/−HTN/−DLP	1.13 (0.64–2.02) *P* = 0.657	1.07 (0.61–1.91) *P* = 0.806	1.07 (0.60–1.90) *P* = 0.817
IFG/+HTN/+DLP	3.24 (2.10–5.01) *P* = 0.000	2.89 (1.86–4.49) *P* = 0.000	2.98 (1.89–4.71) *P* = 0.000
DM/−HTN/−DLP	4.65 (2.75–7.85) *P* = 0.000	4.36 (2.58–7.37) *P* = 0.000	4.51 (2.65–7.68) *P* = 0.000
DM/+HTN/+DLP	4.80 (3.24–7.11) *P* = 0.000	4.46 (2.99–6.63) *P* = 0.000	4.63 (3.03–7.08) *P* = 0.000
(IFG + DM)/−HTN/−DLP	1.97 (1.28–3.03) *P* = 0.002	1.86 (1.21–2.86) *P* = 0.005	1.85 (1.20–2.85) *P* = 0.005
(IFG + DM)/+HTN/+DLP	4.00 (2.83–5.67) *P* = 0.000	3.63 (2.55–5.18) *P* = 0.000	3.64 (2.50–5.31) *P* = 0.000
Category	Unadjusted model	Adjusted model 1	Adjusted model 2^b^
NFG/−HTN	1.0	1.0	1.0
NFG/+ HTN	3.10 (2.35–4.10) *P* = 0.000	2.56 (1.93–3.39) *P* = 0.000	2.68 (2.01–3.56) *P* = 0.000
IFG/−HTN	1.23 (0.80–1.89) *P* = 0.347	1.19 (0.78–1.83) *P* = 0.422	1.20 (0.78–1.85) *P* = 0.400
IFG/+HTN	3.41 (2.44–4.77) *P* = 0.000	2.77 (1.97–3.89) *P* = 0.000	2.88 (2.04–4.07) *P* = 0.000
DM/−HTN	4.27 (2.99–6.12) *P* = 0.000	4.06 (2.84–5.82) *P* = 0.000	4.26 (2.95–6.16) *P* = 0.000
DM/+HTN	4.85 (3.51–6.72) *P* = 0.000	4.13 (2.98–5.74) *P* = 0.000	4.38 (3.10–6.19) *P* = 0.000
(IFG + DM)/−HTN	2.22 (1.62–3.04) *P* = 0.000	2.14 (1.56–2.93) *P* = 0.000	2.15 (1.57–2.95) *P* = 0.000
(IFG + DM)/+HTN	4.04 (3.06–5.35) *P* = 0.000	3.35 (2.52–4.44) *P* = 0.000	3.38 (2.51–4.54) *P* = 0.000
Category	Unadjusted model	Adjusted model 1	Adjusted model 2^c^
NFG/−DLP	1.0	1.0	1.0
NFG/ + DLP	0.99 (0.75–1.31) *P* = 0.953	1.10 (0.83–1.45) *P* = 0.494	1.07 (0.81–1.42) *P* = 0.627
IFG/−DLP	1.18 (0.83–1.68) *P* = 0.358	1.11 (0.78–1.57) *P* = 0.576	1.08 (0.76–1.53) *P* = 0.680
IFG/+DLP	1.35 (0.96–1.89) *P* = 0.084	1.42 (1.01–1.99) *P* = 0.044	1.30 (0.92–1.84) *P* = 0.138
DM/−DLP	2.74 (1.91–3.94) P = 0.000	2.52 (1.75–3.62) P = 0.000	2.35 (1.62–3.41) P = 0.000
DM/+ DLP	2.64 (1.98–3.51) *P* = 0.000	2.70 (2.03–3.61) *P* = 0.000	2.48 (1.83–3.35) *P* = 0.000
(IFG + DM)/−DLP	1.63 (1.23–2.15) *P* = 0.001	1.51 (1.14–2.00) *P* = 0.004	1.44 (1.08–1.91) *P* = 0.012
(IFG + DM)/+DLP	1.93 (1.51–2.48) *P* = 0.000	2.01 (1.56–2.58) *P* = 0.000	1.80 (1.39–2.34) P = 0.000

**Model 1**: adjusted for age + sex. **Model 2**^a^: adjusted for age + sex + BMI + smoking (Never as reference). **Model 2**^b^: adjusted for age + sex + BMI + smoking (Never as reference) + DLP. **Model 2**^c^: adjusted for age + sex + BMI + smoking (Never as reference) + HTN. *NFG* Normal fasting glucose, *IFG* impaired fasting glucose, *HTN* Hypertension, *DLP* Dyslipidemia

**Fig. 1:**
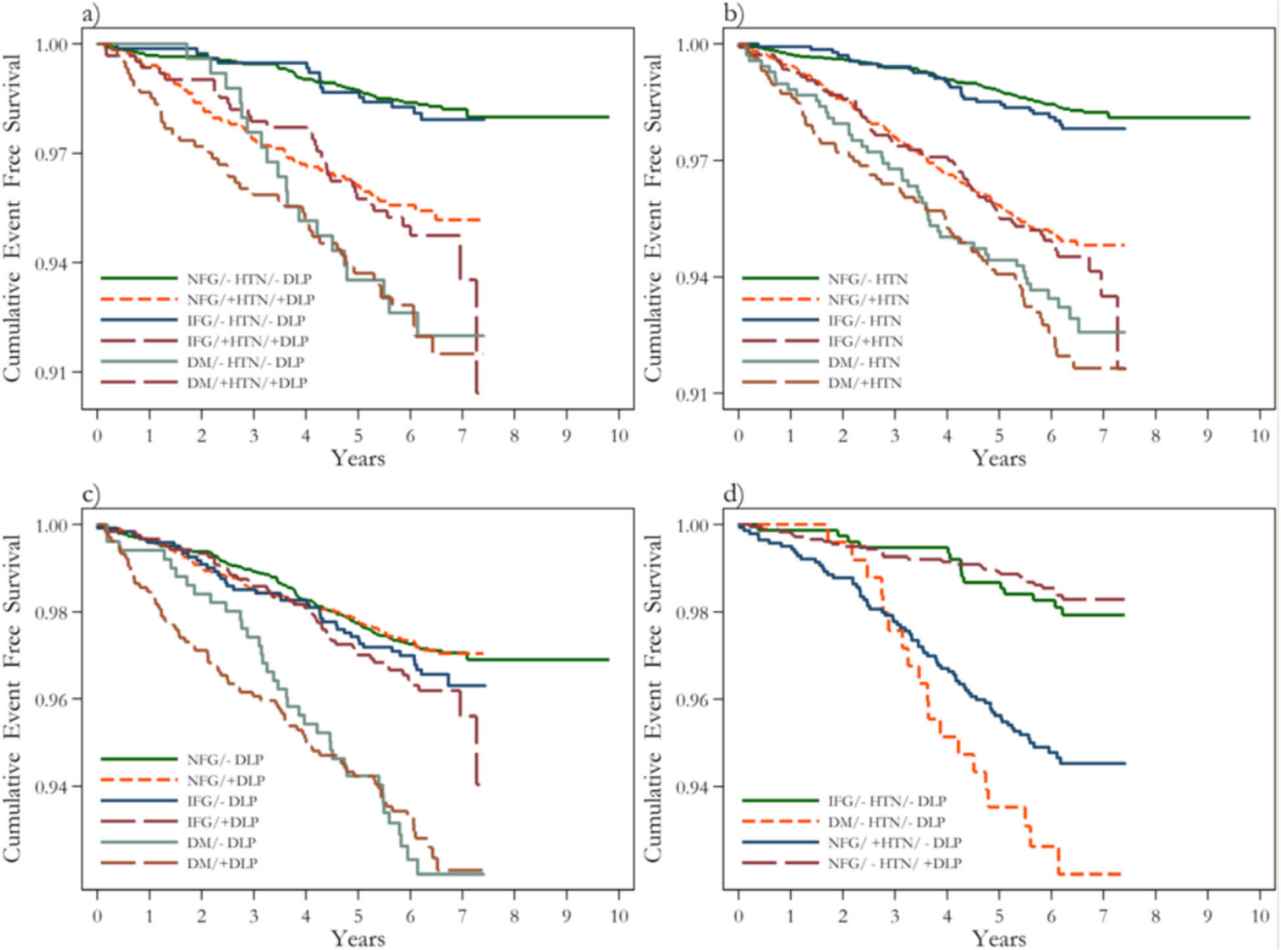
The cumulative event- free survival analysis for occurrence of MACE according to (**a**) glycemic status, HTN, and DLP, (**b**) glycemic status and HTN, (**c**) glycemic status and DLP, (**d**) IFG, DM, HTN, and DLP alone. MACE; major adverse cardiovascular events, NFG; normal fasting glucose, IFG; impaired fasting glucose, DM; diabetes mellitus, HTN; hypertension, DLP; dyslipidemia

Upon adjustment with confounders, comparing to NFG, IFG in the absence of HTN, DLP, or both did not decrease MACE free survival (*p* > 0.05 for all comparisons). Moreover, DM even in the absence of HTN and DLP significantly decreased the MACE free survival (*p* < 0.000 for all comparisons) (Fig. 1).

We next examined the effect of IFG with HTN, DLP, or both HTN + DLP on the occurrence of individual components of MACE in detail. Table 3 shows the effect of IFG in the presence or absence of HTN + DLP on the occurrence of the individual components of MACE defined as non-fatal MI, non-fatal stroke, and CVD-death. IFG in the presence or absence of HTN + DLP did not increase the risk for non-fatal MI compared with NFG in the absence of HTN + DLP (fully adjusted HR, 1.19; 95% CI, 0.25–5.68; p, 0.831) and (fully adjusted HR, 0.87; 95% CI, 0.19–4.02; p, 0.854), respectively. However, IFG in the presence of HTN + DLP was significantly associated with an increase in the risk for non-fatal stroke (fully adjusted HR, 3.18; 95% CI, 1.35–7.47; p, 0.008) and CVD-death (fully adjusted HR, 3.25; 95% CI, 1.84–5.74; p, 0.000). Moreover, DM in the absence or presence of HTN + DLP, as well as IFG + DM in the presence of HTN + DLP significantly increased the risk for non-fatal stroke (fully adjusted HR, 3.44; 95% CI, 1.12–10.59; p, 0.031), (fully adjusted HR, 4.30; 95% CI, 1.90–9.72; p, 0.000), and (fully adjusted HR, 3.61; 95% CI, 1.75–7.45; p, 0.001), respectively. Additionally, DM in the absence or presence of HTN + DLP significantly increased the risk for CVD-death (fully adjusted HR, 5.22; 95% CI, 2.71–10.03; p, 0.000) and (fully adjusted HR, 5.94; 95% CI, 3.55–9.95; p, 0.000), respectively. Furthermore, IFG + DM in the absence or presence of HTN + DLP was significantly associated with an increase in the risk of CVD-death (fully adjusted HR, 2.24; 95% CI, 1.33–3.79; p, 0.003) and (fully adjusted HR, 4.35; 95% CI, 2.73–6.94; p, 0.000), respectively.

**Table 3:** Cox regression models predicting individual components of MACE according to glycemic status, HTN, DLP

Category	HR (95% CI) for incident non-fatal MI		
	Unadjusted model	Adjusted model 1	Adjusted model 2
NFG/−HTN/−DLP	1.0	1.0	1.0
NFG/+HTN/+DLP	3.96 (1.69–9.26) *P* = 0.002	4.34 (1.83–10.28) *P* = 0.001	4.00 (1.63–9.83) *P* = 0.002
IFG/−HTN/−DLP	0.90 (0.20–4.19) *P* = 0.898	0.88 (0.19–4.07) *P* = 0.871	0.87 (0.19–4.02) *P* = 0.854
IFG/+HTN/+DLP	1.18 (0.26–5.48) *P* = 0.830	1.31 (0.28–6.14) *P* = 0.729	1.19 (0.25–5.68) *P* = 0.831
DM/−HTN/−DLP	8.84 (3.15–24.84) *P* = 0.000	8.94 (3.18–25.16) *P* = 0.000	8.45 (2.92–24.46) *P* = 0.000
DM/+HTN/+DLP	1.85 (0.50–6.84) P = 0. 355	2.08 (0.56–7.79) *P* = 0.275	1.88 (0.48–7.32) *P* = 0.364
(IFG + DM)/−HTN/−DLP	2.77 (1.07–7.18) *P* = 0.036	2.72 (1.05–7.05) *P* = 0.040	2.59 (0.99–6.75) *P* = 0.053
(IFG + DM)/+HTN/+DLP	1.51 (0.51–4.51) *P* = 0.460	1.68 (0.55–5.07) *P* = 0.361	1.41 (0.45–4.46) *P* = 0.554
**HR (95% CI) for incident non-fatal stroke**			
Category	Unadjusted model	Adjusted model 1	Adjusted model 2
NFG/−HTN/−DLP	1.0	1.0	1.0
NFG/+HTN/+DLP	1.56 (0.66–3.73) *P* = 0.312	1.40 (0.58–3.37) *P* = 0.453	1. 36 (0.55–3.34) *P* = 0.590
IFG/−HTN/−DLP	0.58 (0.13–2.56) *P* = 0.471	0.55 (0.13–2.43) *P* = 0.431	0.54 (0.12–2.36) *P* = 0.410
IFG/+HTN/+DLP	3.75 (1.66–8.44) *P* = 0.001	3.26 (1.43–7.43) *P* = 0.005	3.18 (1.35–7.47) *P* = 0.008
DM/−HTN/−DLP	3.78 (1.24–11.47) *P* = 0.019	3.52 (1.16–10.70) *P* = 0.027	3.44 (1.12–10.59) *P* = 0.031
DM/+HTN/+DLP	5.07 (2.38–10.79) P = 0.000	4.54 (2.11–9.77) P = 0.001	4.30 (1.90–9.72) P = 0.000
(IFG + DM)/−HTN/−DLP	1.33 (0.51–3.46) *P* = 0.558	1.26 (0.48–3.28) *P* = 0.636	1.22 (0.47–3.18) *P* = 0.687
(IFG + DM)/+HTN/+DLP	4.40 (2.26–8.54) *P* = 0.000	3.86 (1.96–7.62) *P* = 0.000	3.61 (1.75–7.45) *P* = 0.001
**HR (95% CI) for incident CVD- death**			
Category	Unadjusted model	Adjusted model 1	Adjusted model 2
NFG/−HTN/−DLP	1.0	1.0	1.0
NFG/+HTN/+DLP	2.96 (1.82–4.80) P = 0.000	2.62(1.60–4.26) P = 0.000	2.78 (1.68–4.61) P = 0.000
IFG/−HTN/−DLP	1.50 (0.77–2.92) *P* = 0.229	1.40 (0.72–2.72) *P* = 0.321	1.40 (0.72–2.72) *P* = 0.322
IFG/+HTN/+DLP	3.60 (2.09–6.19) *P* = 0.000	3.05 (1.76–5.28) *P* = 0.000	3.25 (1.84–5.74) *P* = 0.000
DM/−HTN/−DLP	3.35 (1.81–10.19) *P* = 0.000	4.91 (2.58–9.37) *P* = 0.000	5.22 (2.71–10.03) *P* = 0.000
DM/+HTN/+DLP	6.14 (3.81–9.89) *P* = 0.000	4.48 (3.38–8.87) *P* = 0.000	5.94 (3.55–9.95) *P* = 0.000
(IFG + DM)/−HTN/−DLP	2.40 (1.42–4.05) *P* = 0.001	2.23 (1.32–3.77) *P* = 0.003	2.24 (1.33–3.79) *P* = 0.003
(IFG + DM)/+HTN/+DLP	4.84 (3.14–7.46) *P* = 0.000	4.20 (2.71–6.52) *P* = 0.000	4.35 (2.73–6.94) *P* = 0.000

**Model 1**: adjusted for age + sex, **Model 2**: adjusted for age + sex + BMI + smoking (Never as reference). *NFG* Normal fasting glucose, *IFG* Impaired fasting glucose, *HTN* Hypertension, *DLP* Dyslipidemia

The results indicated that IFG in the presence or absence of HTN did not increase the risk for non-fatal MI compared to NFG in the absence of HTN (fully adjusted HR, 1.58; 95% CI, 0.56–4.46; p, 0.389) and (fully adjusted HR, 0.71; 95% CI, 0.21–2.46; p, 0.590), respectively. However, IFG in the presence of HTN significantly increased the risk for non-fatal stroke (fully adjusted HR, 2.76; 95% CI, 1.40–5.43; p, 0.003) and CVD-death (fully adjusted HR, 3.12; 95% CI, 2.04–4.78; p, 0.000). Moreover, IFG in the absence of HTN, adjusted for DLP, did significantly increase the risk for CVD-death (fully adjusted HR, 1.65; 95% CI, 1.01–2.68; p, 0.045). Furthermore, DM in the absence or presence of HTN significantly increased the risk for non-fatal stroke (fully adjusted HR, 3.41; 95% CI, 1.59–7.33; p, 0.002) and (fully adjusted HR, 4.47; 95% CI, 2.30–8.69; p, 0.000), respectively. IFG + DM in the presence of HTN was also associated with an increase in the risk for non-fatal stroke (fully adjusted HR, 3.35; 95% CI, 1.89–5.94; p, 0.000). In addition, DM in the absence or presence of HTN significantly increased the risk for CVD- death (fully adjusted HR, 4.10; 95% CI, 3.20–7.81; p, 0.000) and (fully adjusted HR, 5.26; 95% CI, 3.46–7.10; p, 0.000), respectively. The results also indicated a significant increase in the risk for CVD- death in IFG + DM in the absence or presence of HTN (fully adjusted HR, 2.66; 95% CI, 1.82–3.90; p, 0.000) and (fully adjusted HR, 3.84; 95% CI, 2.67–5.53; p, 0.000), respectively (Supplementary Table 1).

IFG in the presence or absence of DLP did not increase the risk for non-fatal MI and non-fatal stroke. However, IFG in the presence of DLP significantly increased the incidence of CVD-death (fully adjusted HR, 1.60; 95% CI, 1.04–2.37; p, 0.032). Moreover, DM in the absence or presence of DLP significantly increased the risk for CVD- death (fully adjusted HR, 2.68; 95% CI, 1.71–4.20; p, 0.000) and (fully adjusted HR, 3.13; 95% CI, 2.18–4.48; p, 0.000), respectively. IFG + DM in the absence or presence of DLP was also significantly associated with an increase in the risk for CVD-death (fully adjusted HR, 1.65; 95% CI, 1.17–2.33; p, 0.004) and (fully adjusted HR, 2.22; 95% CI, 1.62–3.05; p, 0.000), respectively (Supplementary Table 2).

## Discussion

The major findings of the present study on composite CVD outcomes (MACE) in a group of individuals with IFG are that IFG, per se, in the absence of HTN and DLP is not associated with an increase in the risk for MACE. In contrast, IFG in the presence of HTN or HTN + DLP is associated with an incremental and significant increase in the risk for MACE. Further analyses regarding the impact of IFG in the presence of HTN, DLP, or HTN + DLP on the individual components of MACE demonstrated that IFG in the presence of HTN or HTN + DLP incrementally increases the risk for non-fatal stroke and CVD-death. Additionally, the combination of IFG with DLP is associated with an increase in the risk for CVD-death. As expected, DM in the absence or presence of HTN, DLP, or both is significantly associated with an increase in the risk for MACE. Moreover, pooled analysis of IFG + DM shows dysglycemic status compared with normoglycemic condition increases the risk for MACE even in the absence of other CV risk factors such as HTN and DLP.

Whether pre-DM, per se, is an independent risk factor for CVD has been a matter of debate. In the current study, IFG in the absence of other CV risk factors did not increase the risk for composite end-point of MACE. However, IFG in the absence of HTN, adjusted for DLP, significantly increased the risk for CVD- death. Likewise, other longitudinal population-based studies, also did not find any association between pre-DM alone and CVD risk [14, 15]. Moreover, Barr et al. showed IFG is an independent predictor for CVD mortality [16]. However, some studies have indicated that dysglycemia within pre-diabetic glucose range was associated with an increase in the risk for CVD [6, 17, 18]. Most studies have suggested that impaired glucose tolerance (IGT) is a stronger predictor than IFG for CVD events [19, 20]. But, a recently published study demonstrated the incidence of MACE in patients with established coronary artery disease (CAD) and pre-DM, defined as either of IFG or IGT, does not differ from those in patients with normal glycemic status [21]. Furthermore, a previous study conducted to assess the risk of death according to the different diagnostic glucose categories showed “fasting blood glucose alone is not sufficient to predict mortality related to hyperglycemia” They found in any category of fasting glucose, an increase in the 2-h post-load glucose is associated with a higher risk of mortality [22]. Likewise, results from a recent review suggested that IGT and HbA1C correlate more with CVD risk than IFG [23]. These inconsistencies might be due to differences in analytical methods, different definitions for pre- diabetes, and ethnic origins of the participants included in the studies.

It is generally agreed that individuals with pre-diabetes or T2DM have higher than normal CV risk factors. This is probably due to the fact that T2DM and pre-DM share common underlying pathophysiologic disturbances such as insulin resistance [2]. In our study, as expected, participants with IFG had significantly higher BMI, WC, SBP, DBP, TG, total cholesterol, and LDL compared to normoglycemic subjects. Moreover, they were more likely to have HTN and DLP than those with NFG. Similar results are frequently reported in previous studies investigating the presence of CV risk factors in individuals with pre-DM [9, 10, 24].

Aggregation of CV risk factors in individuals with pre-DM is known to place them at higher risk for CV events [10]. In the current study we found that IFG when associated with HTN significantly increased the risk of composite end-point of MACE. This result is in line with the results of some previous studies in which the coexistence of pre-DM and HTN, but not pre-DM without HTN, significantly increased the risk of CVD events [14, 15]. Another study showed that pre-DM defined as IFG or IGT even in the presence of prehypertension, defined as SBP between120 to 139 or DBP between 80 to 89 mmHg, significantly increases the risk for CVD events [25]. HTN is a well-known and powerful risk factor for almost all different types of CVD events [26]. Similarly, the current analysis indicated HTN alone in normoglycemic participants is associated with higher HRs for both MACE and all individual components of MACE. The reason why coexistence of pre-DM and HTN, but not pre-DM without HTN, leads to higher CVD events might reflect the fact that both of them induce endothelial dysfunction and inflammation through elevation of inflammatory factors such as tumor necrosis factor α (TNF α) and intercellular adhesion molecule-1 (ICMA-1) [27]. Hence, co-occurrence of HTN, or even pre-HTN, with pre-DM results in higher levels of inflammatory factors and more severe endothelial damage conferring additive risk for subsequent occurrence of composite end-point of MACE.

DLP, characterized by elevated TG, low concentration of HDL, and an increased number of small dense LDL, plays a critical role in acceleration of atherosclerosis and is reported as an important independent risk factor for CVD [28]. Several studies have demonstrated that individuals with pre-diabetes have deranged lipid profiles compared to normoglycemic subjects [9, 22]. The frequent association of DLP with pre-DM, which may reflect the underlying insulin resistance, has led to frequent treatment of DLP in the pre-diabetes [29, 30]. Despite of the importance of screening and treatment of DLP in individuals with pre-diabetes, we have not found studies that combined pre-DM and DLP together in the analysis for predicting CVD risk. Nevertheless, when we compared the impact of IFG plus DLP with that of NFG without DLP, we found that coexistence of DLP and IFG did not confer any additional risk for development of the composite end-point of MACE. One possible explanation for this finding is that individuals with IFG were more likely to use statin compared to normoglycemic individuals. Nowadays, statin therapy is widely recommended for both primary and secondary prevention of CV disease in a wide range of individuals, even in those without hyperlipidemia [31, 32]. Statins exert their beneficial effects not only through lowering of LDL, but also by improving endothelial function [33], reducing vascular inflammation [32, 34], and exerting antithrombotic actions [35].

With regard to the addition of both HTN + DLP to IFG, the present analyses showed an incremental increase in the occurrence of composite end-point of MACE. Although addition of DLP to IFG did not increase the risk for MACE, co-existence of DLP + HTN increased the risk for MACE even more than addition of HTN alone to IFG. Our findings support the results of previous studies showing that the risk of CV events associated with co-existence of HTN and DLP, labeled as “lipitation”, is greater than the sum of the CV risks for HTN and DLP alone [36]. The interaction between these two risk factors occurs at the vascular endothelial level and results in increased oxidative stress and endothelial dysfunction, disproportionate vascular contractility, elevation of BP in dyslipidemic patients, further elevation of BP in hypertensive patients, and accelerates the development and progression of atherosclerotic lesions [37]. A similar finding has been reported in a large recent study conducted among patients with 5 years’ duration of T2DM. The results of this study showed that patients with T2DM who had no CV risk factors had little or no excess risk of CVD events or death compared with general population with no diabetes [38]. The findings highlight the significance of control of CV risk factors both in patients with pre-DM and T2DM.

Considering the effect of IFG combined with CV risk factors on the individual components of MACE, we found that IFG in the absence of HTN, adjusting for DLP, significantly increased the risk for CVD-death. Addition of HTN, or HTN + DLP to IFG was associated with an incremental increase in the risk for non-fatal stroke and CVD-death. In addition, IFG combined with DLP alone significantly increased the risk for CVD- death. The reason why IFG in the absence of HTN, but not in the absence of both HTN and DLP did increase the risk for CVD-death might be explained by the confounding effect of medications such as statin that has been previously shown to be independently associated with a decrease in the risk for CVD mortality [39].

As expected DM either alone or in combination with HTN, DLP, or HTN + DLP significantly increased the risk for composite end-pint of MACE as well as the individual components of non-fatal stroke and CVD- death. However, addition of HTN, DLP, or HTN+ DLP to DM did not increase the risk for non-fatal MI. These inconclusive findings could be due to the small number of events in each group. In addition, further use of statin might protect participants with DM who also have HTN, DLP, or HTN + DLP from some individual CV events.

Moreover, dysglycemia (IFG + DM) either alone or combined with HTN, DLP, or HTN+ DLP was associated with an increase in the risk for MACE. This finding highlighted the fact that high glucose level of any degree might have a significant adverse effect on CV outcomes.

This study has several strengths. This study investigated the impact of IFG alone or combined with other well-established CV risk factors such as HTN, DLP, or HTN + DLP, on the occurrence of MACE. We included an adequately large number of participants without any known CVD at baseline and the study had 99% follow-up of participants. In addition, we adjusted for multiple confounding factors including most of the traditional CVD risk factors. Finally, the occurrence of individual CV outcomes, including non-fatal MI, non-fatal stroke, and CVD-death, was assessed. Weaknesses include the fact that, due to non-availability of 2 h post-prandial blood glucose, the effect of only IFG as a component of pre-diabetes could be examined. Although evidence shows that IFG is useful for screening of pre-diabetes in the general population, use of IGT and HbA1C might be stronger predictors of cardiovascular events [20, 40]. Finally, considering the study design, the findings might be subjected to unmeasured confounding factors. In addition, risk factors were only measured at baseline. Therefore, the impact of the changes could not be evaluated.

## Conclusion

In summary, although IFG, per se, did not increase the risk for MACE, the association of IFG with HTN or HTN+ DLP conferred a significant risk for composite end-point of MACE and some individual components such as non-fatal stroke and CVD- death in an incremental manner. Moreover, IFG alone in the absence of HTN, adjusted for DLP, significantly increased the risk for CVD- death. These findings support the idea that emphasis should be placed on identification and treatment of multiple CV risk factors in people with pre-diabetes.

## Supplementary information

**Additional file 1 Supplementary Table 1** Cox models predicting components of MACE according to glycemic status and HTN. **Supplementary Table 2** Cox regression models predicting individual components of MACE according to glycemic status and DLP

## Data Availability

The datasets used and/or analyzed during the current study are available from the corresponding author on reasonable request.

## Abbreviations

ADA: American diabetes association criteria
BMI: Body mass index
BP: Blood pressure
CV: Cardiovascular
CVD: Cardiovascular disease
DBP: Diastolic blood pressure
DLP: Dyslipidemia
FBS: Fasting blood glucose
GCS: Golestan cohort study
HDL: High density lipoprotein
HTN: Hypertension
IFG: Impaired fasting glucose
IGT: Impaired glucose tolerance
LDL: Low density lipoprotein
MACE: Major adverse cardiovascular events
NFG: Normal fasting glucose
Pre-DM: Pre-diabetes
SBP: Systolic blood pressure
T2DM: Type 2 diabetes
TG: Triglyceride
WC: Waist circumference
