# Supplementary material for: Impaired fasting glucose and major adverse cardiovascular events by hypertension and dyslipidemia status: the Golestan cohort study
Source: BMC Cardiovasc Disord. 2020 Mar 5;20:113. doi: 10.1186/s12872-020-01390-8 (PMC7057517; doi:10.1186/s12872-020-01390-8)
Supplement: Supplementary file 1 — Additional file 1 Supplementary Table 1 Cox models predicting components of MACE according to glycemic status and HTN. Supplementary Table 2 Cox regression models predicting individual components of MACE according to glycemic status and DLP [file 12872_2020_1390_MOESM1_ESM.docx]

**Supplementary Table 1** Cox models predicting components of MACE according to glycemic status and HTN

|  | HR (95% CI) for incident non-fatal MI | | | |  |
| --- | --- | --- | --- | --- | --- |
| Category | Unadjusted model | Adjusted model 1 | Adjusted model 2 |  |  |
| NFG/-HTN | 1.0 | 1.0 | 1.0 |  |  |
| NFG/+HTN | 2.65 ( 1.36- 5.18)  P= 0.004 | 2.61 (1.32- 5.17)  P= 0.006 | 2.60 ( 1.30- 5.20)  P= 0.007 |  |  |
| IFG/-HTN | 0.72 (0.21-2.47)  P=0.597 | 0.71 (0.21-2.46)  P=0.589 | 0.71 (0.21-2.46)  P=0.590 |  |  |
| IFG/+HTN | 1.59 (0.58-4.38)  P=0.369 | 1.60 (0.57-4.43)  P=0.370 | 1.58 (0.56-4.46)  P=0.389 |  |  |
| DM/-HTN | 4.00 (1.70- 9.44)  P= 0.002 | 4.06 (1.72- 9.58)  P= 0.001 | 4.02 (1.66- 9.74)  P= 0.002 |  |  |
| DM/ +HTN | 2.44 (0.95- 6.29)  P= 0.065 | 2.49 (0.96- 6.49)  P= 0.062 | 2.46 (0.91- 6.68)  P= 0.076 |  |  |
| (IFG+DM)/-HTN | 1.78 (0.82-3.87)  P=0.147 | 1.77 (0.81-3.87)  P=0.149 | 1.73 (0.79-3.81)  P=0.170 |  |  |
| (IFG+DM)/+HTN | 1.96 (0.90-4.27)  P=0.089 | 1.97 (0.89-4.35)  P=0.092 | 1.87 (0.83-4.26)  P=0.133 |  |  |
|  | | | | | |
|  | HR (95% CI) for incident non-fatal stroke | | | |  |
| Category | Unadjusted model | Adjusted model 1 | Adjusted model 2 |  |  |
| NFG/-HTN | 1.0 | 1.0 | 1.0 |  |  |
| NFG/+HTN | 2.51 ( 1.43- 4.38)  P= 0.001 | 2.06 ( 1.17- 3.62)  P= 0.013 | 2.13 (1.20-3.79)  P= 0.010 |  |  |
| IFG/-HTN | 0.65 (0.22-1.89)  P=0.432 | 0.64 (0.22-1.85)  P=0.405 | 0.64 (0.22-1.86)  P=0.415 |  |  |
| IFG/+HTN | 3.22 (1.67-6.21)  P=0.000 | 2.61 (1.34-5.08)  P=0.005 | 2.76 (1.40-5.43)  P=0.003 |  |  |
| DM/- HTN | 3.40 (1.61- 7.17)  P= 0.001 | 3.21 (1.52- 6.79)  P= 0.002 | 3.41 (1.59- 7.33)  P= 0.002 |  |  |
| DM/+ HTN | 4.92 ( 2.64- 9.17)  P= 0.000 | 4.13 ( 2.20- 7.75)  P= 0.000 | 4.47 (2.30- 8.69)  P= 0.000 |  |  |
| (IFG+DM)/-HTN | 1.54 (0.79-3.02)  P=0.205 | 1.49 (0.76-2.91)  P=0.244 | 1.50 (0.76-2.95)  P=0.238 |  |  |
| (IFG+DM)/+HTN | 3.97 (2.31-6.81)  P=0.000 | 3.26 (1.88-5.64)  P=0.000 | 3.35 (1.89-5.94)  P=0.000 |  |  |
|  | | | | | |
| HR (95% CI) for incident CVD-death | | | | | |
| Category | Unadjusted model | Adjusted model 1 | Adjusted model 2 |  |  |
| NFG/-HTN | 1.0 | 1.0 | 1.0 |  |  |
| NFG/+HTN | 3.27 ( 2.30- 4.65)  P= 0.000 | 2.58 ( 1.81- 3.69)  P= 0.000 | 2.73 (1.90-3.92)  P= 0.000 |  |  |
| IFG/-HTN | 1.70 (1.05-2.77)  P=0.032 | 1.64 (1.01-2.67)  P=0.047 | 1.65 (1.01-2.68)  P=0.045 |  |  |
| IFG/+HTN | 3.86 (2.55-5.83)  P=0.000 | 2.99 (1.97-4.54)  P=0.000 | 3.12 (2.04-4.78)  P=0.000 |  |  |
| DM/- HTN | 5.05 (3.26- 7.80)  P= 0.000 | 4.73 (3.06- 7.33)  P= 0.000 | 4.10 (3.20- 7.81)  P= 0.000 |  |  |
| DM/+ HTN | 6.01 (4.06- 8.90)  P= 0.000 | 4.93 (3.22- 7.33)  P= 0.000 | 5.26 (3.46- 7.10)  P= 0.000 |  |  |
| (IFG+DM)/-HTN | 2.78 (1.91-4.06)  P=0.000 | 2.66 (1.82-3.88)  P=0.000 | 2.66 (1.82-3.90)  P=0.000 |  |  |
| (IFG+DM)/+HTN | 4.80 (3.40-6.78)  P=0.000 | 3.81 (2.69-5.41)  P=0.000 | 3.84 (2.67-5.53)  P=0.000 |  |  |

**Model 1**: adjusted for age + sex, **Model 2**: adjusted for age + sex + BMI + smoking (Never as reference) + DLP. MACE; major adverse cardiovascular events, NFG; normal fasting glucose, IFG; impaired fasting glucose, HTN; hypertension, DLP; dyslipidemia.

**Supplementary Table 2** Cox regression models predicting individual components of MACE according to glycemic status and DLP

|  | HR (95% CI) for incident non-fatal MI | | |
| --- | --- | --- | --- |
| Category | Unadjusted model | Adjusted model 1 | Adjusted model 2 |
| NFG/- DLP | 1.0 | 1.0 | 1.0 |
| NFG/+DLP | 1.82 ( 0.93- 3.53)  P= 0.079 | 1.97 ( 1.01- 3.85)  P= 0.047 | 1.84 ( 0.93- 3.63)  P= 0.078 |
| IFG/- DLP | 1.15 (0.42-3.14)  P=0.785 | 1.13 (0.41-3.09)  P=0.811 | 1.10 (0.40-3.00)  P=0.856 |
| IFG/+ DLP | 0.70 (0.21-2.41)  P=0.576 | 0.76 (0.22-2.60)  P=0.659 | 0.67 (0.19-2.33)  P=0.528 |
| DM/- DLP | 5.24 (2.32-11.88)  P= 0.000 | 5.20 (2.29- 11.80)  P= 0.000 | 4.64 (2.00- 10.75)  P= 0.000 |
| DM/+ DLP | 1.42 (0.52- 3.88)  P= 0.493 | 1.52 ( 0.55- 4.15)  P= 0.418 | 1.30 ( 0.46- 3.64)  P= 0.620 |
| (IFG+DM)/- DLP | 2.31 (1.13-4.73)  P=0.022 | 2.27 (1.11-4.66)  P=0.025 | 2.12 (1.03-4.37)  P=0.042 |
| (IFG+DM)/+ DLP | 1.03 (0.44-2.40)  P=0.949 | 1.10 (0.47-2.58)  P=0.829 | 0.93 (0.39-2.21)  P=0.862 |
|  |  | | |
|  | HR (95% CI) for incident non-fatal stroke | | |
| Category | Unadjusted model | Adjusted model 1 | Adjusted model 2 |
| NFG/- DLP | 1.0 | 1.0 | 1.0 |
| NFG/+DLP | 0.72 (0.40- 1.30)  P= 0.274 | 0.78 ( 0.43-1.41)  P= 0.404 | 0.75 ( 0.41-1.36)  P= 0.341 |
| IFG/- DLP | 0.76 (0.34-1.71)  P=0.508 | 0.72 (0.32-1.62)  P=0.428 | 0.70 (0.31-1.58)  P=0.386 |
| IFG/+ DLP | 1.32 (0.68-2.55)  P=0.409 | 1.35 (0.70-2.603)  P=0.366 | 1.22 (0.62-2.40)  P=0.558 |
| DM/- DLP | 2.47 (1.18- 5.14)  P= 0.016 | 2.26 ( 1.08- 4.71)  P= 0.030 | 2.09 (0.99- 4.41)  P= 0.054 |
| DM/+DLP | 2.51 (11.43- 4.41)  P= 0.001 | 2.50 ( 1.42- 4.40)  P= 0.001 | 2.22 ( 1.23- 4.02)  P= 0.008 |
| (IFG+DM)/- DLP | 1.24 (0.69-2.25)  P=0.471 | 1.17 (0.64-2.11)  P=0.613 | 1.10 (0.61-2.01)  P=0.746 |
| (IFG+DM)/+ DLP | 1.86 (1.14-3.03)  P=0.012 | 1.88 (1.15-3.08)  P=0.012 | 1.65 (0.99-2.75)  P=0.056 |
|  | | | |
| HR (95% CI) for incident CVD-death | | | |
| Category | Unadjusted model | Adjusted model 1 | Adjusted model 2 |
| NFG/- DLP | 1.0 | 1.0 | 1.0 |
| NFG/+DLP | 0.99(0.70- 1.40)  P= 0.941 | 1.12( 0.79-1.60)  P= 0.511 | 1.12( 0.78-1.59)  P= 0.541 |
| IFG/- DLP | 1.39 (0.92-2.11)  P=0.116 | 1.29 (0.85-1.95)  P=0.226 | 1.27 (0.84-1.92)  P=0.266 |
| IFG/+ DLP | 1.55(1.04-2.33)  P=0.030 | 1.66 (1.11-2.49)  P=0.013 | 1.60 (1.04-2.37)  P=0.032 |
| DM/- DLP | 3.08 (1.10- 4.77)  P= 0.000 | 2.79 ( 1.80- 4.32)  P= 0.000 | 2.68 ( 1.71- 4.20)  P= 0.000 |
| DM/+DLP | 3.15 (2.25- 4.43)  P= 0.000 | 3.27 ( 2.32- 4.60)  P= 0.000 | 3.13 ( 2.18- 4.48)  P= 0.000 |
| (IFG+DM)/- DLP | 1.87 (1.34-2.62)  P=0.000 | 1.72 (1.23-2.41)  P=0.002 | 1.65 (1.17-2.33)  P=0.004 |
| (IFG+DM)/+ DLP | 2.28 (1.69-3.08)  P=0.000 | 2.40 (1.77-3.25)  P=0.000 | 2.22 (1.62-3.05)  P=0.000 |

**Model 1**: adjusted for age + sex, **Model 2**: adjusted for age + sex + BMI + smoking (Never as reference) + HTN. MACE; major adverse cardiovascular events, NFG; normal fasting glucose, IFG; impaired fasting glucose; HTN; hypertension, DLP; dyslipidemia.
